# Treatise on Internal Hydrocephalus

**Published:** 1841-07

**Authors:** 


ART. X.—*Traité sur l'Hydrocephale Intérne.* Par G. VROLIK, Professeur à l'Athénée d'Amsterdam, &c.—*Amsterdam,* 1839. 4to, pp. 11. Pl. 3.

*Treatise on Internal Hydrocephalus.* By G. VROLIK, Professor at the Atheneum at Amsterdam, &c.—*Amsterdam,* 1839. 4to, pp. 11, with 3 Plates.

THIS brief memoir contains several interesting observations. The author believes that such an hydrocephalus as can be referred to an arrest of normal development is very rare; and, maintaining his opinion chiefly by the fact of the frequent occurrence of false membranes limiting the effusion, holds that the great majority of cases have an inflammatory origin. He remarks on the differences of form assumed by the head according to the situations in which effusion has taken place. When the fluid collects at once in all the ventricles, the expansion of the skull is uniform; but he possesses the skull of a girl which is very much larger on the left side of the base than on the right, in consequence of a circumscribed dropsy of the middle horn of the left ventricle. And again, he has sometimes observed, that when the effusion is limited to the lateral ventricles, or to them and the third, then the expansion is confined to the bones above the occipital, and the latter is scarcely at all increased in size.

Our own observations, as well we believe as Dr. Foville's, are confirmatory of the statements of Dr. Vrolik; and we can add that peculiar forms are sometimes presented by hydrocephalic heads, in consequence of unnatural closure of the sutures preventing the expansion of particular parts of the skull. An illustration of this is now before us, in the skull of a subject that died at about twelve years old; the sagittal suture is completely closed, and the head, expanded in every direction except the transverse, has assumed a singularly elongated form, with a very high elevated but narrow forehead.

Professor Vrolik remarks also on the fact that the more slowly the effusion takes place and the head expands, the less danger is there of loss either of life or of intellect; for in these cases it is that the brain, though it changes its form, retains its structure quite unaltered. He has the skeleton of a man who died hydrocephalic at the age of sixty. He was only four feet four inches high, having been extremely rickety; but his skull measures 1·85 feet in circumference, and 1·35 feet from the nose over the vertex to the foramen magnum. The other dimensions are well proportioned to these, yet during life the individual had exhibited a cultivated mind and a highly-developed intellect.

The author fully agrees in the opinion of Gall respecting the *unfolding* of the convolutions in hydrocephalus; and points out that the fact was known by Hernauld, who described it, though he could not explain it, in the "Histoire de l'Académie Royale des Sciences, 1740," p. 375. Hitherto, however, the fact has not been well illustrated; and therefore, to supply this deficiency, the author annexes three engravings from two cases of cerebral expansion or unfolding which he met with in 1812, and all of which show in a remarkable manner the almost complete obliteration of all appearance of convolutions or irregularities on the surface of the brain. At the same time, however thin the layer of cerebral substance thus expanded, he observes that every portion of it showed its normally distinct constitution of white and gray matter, a fact which, with the others more commonly noticed, can leave no doubt that the change which the brain undergoes in simple hydrocephalus is one of form only, and that both its structures and (if the effusion have taken place slowly) its functions may remain unaltered. All these skulls moreover present examples of unequal expansion, in consequence of the unequal accumulation of the fluid in the several ventricular cavities.

